# Hyperlactatemia, Coagulopathy, and Hepatic Injury as Prognostic Markers of Mortality in Pediatric Dengue: A Single-Center Retrospective Cohort Study

**DOI:** 10.7759/cureus.101498

**Published:** 2026-01-13

**Authors:** Shikha Swaroop, Ratan Kumar, Preeti Srivastava, Adyasha Mishra, Sanjay K Tanti, Bibekananda Mukherjee, Rishi Anand

**Affiliations:** 1 Department of Pediatrics, Tata Main Hospital, Jamshedpur, IND; 2 Department of Pediatrics, Manipal Tata Medical College, Manipal Academy of Higher Education (MAHE), Jamshedpur, IND; 3 Department of Anesthesiology, Manipal Tata Medical College, Manipal Academy of Higher Education (MAHE), Jamshedpur, IND; 4 Department of Anesthesiology, Tata Main Hospital, Jamshedpur, IND

**Keywords:** acute dengue, aspartate amino transferase, pediatric mortality, predictive model, prothrombin time (pt), serum lactate

## Abstract

Background: Severe dengue has a high mortality rate, especially when identification is delayed. Objective, early laboratory discriminators of mortality could improve triage and resource allocation in pediatric critical care. This study aimed to identify robust admission laboratory discriminators of mortality among children with dengue and to evaluate their individual prognostic performance.

Methodology: We conducted a single-center retrospective observational study of 114 pediatric patients with confirmed dengue infection admitted to the pediatric critical care unit of Tata Main Hospital, Jamshedpur, between June 2023 and December 2023. Admission to the unit followed a uniform institutional protocol based on World Health Organization (WHO) guidelines. Demographic, clinical, and laboratory data (including lactate levels, coagulation profiles, and liver enzymes) were extracted from electronic records. Statistical analyses included the Mann-Whitney U test for continuous variables, Fisher’s exact test for categorical variables, Spearman’s correlation, and receiver operating characteristic (ROC) analysis.

Results: Non-survivors (*n *= 15, 13.15%) had significantly higher lactate (13.6 vs. 3.0 mmol/L, *P *< 0.001), prothrombin time-international normalized ratio (PT-INR) (2.48 vs. 1.25, *P *< 0.001), and aspartate aminotransferase (AST) (1185.0 vs. 215.4 U/L, *P *= 0.003) compared to survivors. ROC analysis demonstrated strong individual discriminative ability: lactate AUC = 0.941 (95% confidence interval (CI): 0.898-0.984), PT-INR AUC = 0.918 (0.865-0.971), AST AUC = 0.743 (0.602-0.884). Data-driven Youden cutoffs were as follows: lactate ≥6.6 mmol/L (sensitivity 93.3%, specificity 90.9%), PT-INR ≥1.855 (sensitivity 86.7%, specificity 89.9%), and AST ≥841.5 U/L (sensitivity 73.3%, specificity 78.8%). Strong positive correlations were observed between these parameters (lactate vs. PT-INR: ρ = 0.585, *P *< 0.001).

Conclusions: Hyperlactatemia, coagulopathy, and hepatocellular injury are significantly correlated with mortality in dengue, with lactate and PT-INR demonstrating particularly robust individual discriminative capabilities. These readily available biomarkers may help identify high‑risk patients early, but the proposed cutoffs require prospective multicenter validation before routine integration into dengue management protocols.

## Introduction

Dengue fever, a mosquito-borne viral infection, has seen a significant increase in incidence and prevalence both globally and in India. Dengue virus infection affects approximately 390 million people annually worldwide, with an estimated 96 million manifesting clinically significant disease [1]. Severe dengue, particularly in the form of dengue hemorrhagic fever (DHF) and dengue shock syndrome (DSS), is a critical concern because of its potential to cause life-threatening complications, such as plasma leakage, hemorrhage, and organ impairment [2]. Prolonged coagulation, renal failure, transaminitis, extremely low platelet counts, and multiorgan dysfunction associated with severe dengue can lead to mortality, as documented in cohort studies. The case fatality rate for severe dengue is 2%-5% in many endemic regions, rising significantly with delayed medical care [3]. With appropriate intervention, it can be reduced from over 20% to <1% [4]. The World Health Organization (WHO) classifies dengue into three groups: dengue without warning signs, dengue with warning signs, and severe dengue [5]. While this classification emphasizes warning signs such as abdominal pain, persistent vomiting, and fluid accumulation, these clinical signs often appear late, limiting early intervention. Laboratory biomarkers that predict adverse outcomes early can improve patient triage and resource allocation.

Several studies have investigated the potential predictors of severe dengue, including platelet counts [6], hematocrit changes [7], and liver transaminase levels [8]. However, the role of metabolic parameters, such as lactate levels and comprehensive coagulation profiles, remains underexplored. In this context, there is a need for simple, objective admission biomarkers that can refine risk stratification beyond clinical warning signs in pediatric dengue. This retrospective cohort study aimed to identify determinants of mortality in dengue infection using non-parametric statistical methods, focusing on patients' metabolic, hepatic, and coagulation lab parameters. The objective was to assess the prognostic performance of three biomarkers representing the 'fatal triad' of severe dengue pathogenesis: serum lactate (indicating tissue hypoperfusion and shock), prothrombin time (measuring coagulopathy and endothelial activation), and liver transaminase (evaluating viral hepatic tropism and ischemic injury) [9-11]. We aimed to determine if admission values of these parameters could serve as early discriminators of mortality in a pediatric critical care cohort.

## Materials and methods

Study design, setting, and population

This retrospective, single-center, observational cohort study was conducted at Tata Main Hospital, Jamshedpur, a tertiary care referral center in an endemic region of India, from June 2023 to December 2023, encompassing the entire seasonal epidemic wave. A convenience sampling method was utilized, enrolling all patients meeting the inclusion criteria within the defined study period. Ethical approval and the requirement for informed consent were waived by the Institutional Ethics Committee, as this was a retrospective study using de-identified clinical data.

We included patients aged 1 month to 16 years, admitted to the pediatric critical care unit (PICU) with confirmed dengue, defined by the WHO criteria (positive nonstructural protein 1 (NS1) antigen, immunoglobulin M (IgM) serology, or polymerase chain reaction (PCR)). Admission to the PICU followed a uniform institutional protocol. Patients were eligible for inclusion if they met the protocol criteria for severe illness, specifically: evidence of hemodynamic instability (shock), presence of clinical warning signs, risk of severe bleeding, respiratory distress due to plasma leakage, or multi-organ dysfunction. The exclusion criteria were incomplete medical records, an alternative primary diagnosis, or transfer from another hospital >24 hours after pediatric critical care admission, to ensure capture of early admission parameters.

Data collection

Data were extracted from the electronic medical records using a standardized form. To ensure that the data accurately represented the acute severity of illness at presentation, all laboratory parameters analyzed were the initial values obtained within 24 hours of PICU admission. The variables included demographics (age and sex), clinical parameters (fever duration before admission, shock, and acute kidney injury (AKI)), and laboratory values. AKI was defined according to serum creatinine and/or urine output criteria. All laboratory results were the first available within 24 hours of admission and included a complete blood count, liver enzymes alanine aminotransferase (ALT) and aspartate aminotransferase (AST), a coagulation profile with prothrombin time-international normalized ratio (PT-INR), venous blood gas parameters (serum lactate, bicarbonate, and pH), and renal function tests. Blood samples were collected at PICU via a standardized venous sampling institutional protocol to ensure uniformity and minimize pre-analytical errors (e.g., prolonged tourniquet application).

Statistical analysis

Continuous variables were assessed for normality using the Shapiro-Wilk test. For non-normal distribution, non-parametric tests were applied. Continuous variables are reported as medians with interquartile ranges (IQRs), while categorical variables are presented as counts and percentages. To investigate associations between admission parameters and mortality, the Mann-Whitney U test was used for continuous variables and Fisher's exact test for categorical variables, given the limited sample size in the non-survivor group. The analysis assessed univariable associations between admission parameters and mortality using these statistical tests. After identifying significant markers, further testing was conducted to determine their prognostic utility. Spearman's correlation coefficient (ρ) was used to assess relationships between lab parameters. To assess the discriminative capacity of the selected biomarkers, receiver operating characteristic (ROC) curve analysis was performed. As this was a derivation study aimed at hypothesis generation, Youden’s index (J = sensitivity + specificity - 1) was calculated to mathematically determine the optimal cutoff values that maximize differentiation between survivors and non-survivors. ROC analysis was conducted to determine the area under the curve (AUC) with 95% confidence intervals (CIs) and identify optimal cutoffs using Youden's index (J = sensitivity + specificity - 1) for individual biomarkers. Pairwise AUC comparisons were made using DeLong's test. These data-driven cutoff values were further assessed for clinical relevance and in the context of previous studies on severe organ dysfunction [9,10]. A *P*-value < 0.05 was considered statistically significant. Statistical analyses were performed using Jamovi statistical software (The Jamovi Project, 2025, Version 2.6).

## Results

Patient characteristics

The cohort included 114 patients, comprising 15 non-survivors (13.15%) and 99 survivors (86.85%). Non-survivors were younger than survivors (96 vs. 128 months, *U* = 464, *P* = 0.02) and had significantly higher rates of shock (100% vs. 5.1%, *P *< 0.001) and AKI (73.3% vs. 15.2%, *P *< 0.001). There were no significant differences in sex distribution or fever duration on admission between the groups (Table 1). The demographic and clinical characteristics of the patients are summarized in Tables 1-2.

**Table 1:** Baseline characteristics of the study population (continuous variables). *P-value from the Mann-Whitney U test. IQR, interquartile range; SD, standard deviation

Variable	Overall (*N* = 114)	Survivors (*n* = 99)	Non-survivors (*n* = 15)	*P*-value
Age (months), Median (IQR)	120 (86.75-157.5)	128 (94-162)	96 (66-114)	0.02*
Fever duration (days), Median (IQR)	4 (3-5)	4 (2-4)	4 (3-5)	0.67*

**Table 2:** Baseline characteristics of the study population (categorical variables). ^‡^*P*-value from Fisher’s exact test.

Variable	Category	Overall (*N* = 114)	Survivors (*n* = 99)	Non-survivors (*n* = 15)	*P*-value
Sex, *n* (%)	Male	75 (65.8)	64 (56.1)	11 (9.6)	0.555^‡^
	Female	39 (34.2)	35 (30.7)	4 (3.5)	
Shock, *n* (%)	Present	20 (17.5)	5 (5.1)	15 (100)	<0.001^‡^
	Absent	94 (82.5)	94 (94.9)	0 (0)	
Acute kidney injury, *n* (%)	Present	26 (22.8)	15 (15.2)	11 (73.3)	<0.001^‡^
	Absent	88 (77.2)	84 (84.8)	4 (26.7)	

Laboratory parameters

At admission, non‑survivors exhibited markedly higher lactate, AST, ALT, PT, PT‑INR, and creatinine levels than survivors, while platelet counts were similarly low in both groups (Table 3).

**Table 3:** Admission laboratory parameters. *P*-value from the Mann-Whitney U test. IQR, interquartile range; U value, Mann-Whitney U statistics

Parameter	Survivors (*n* = 99)	Non-survivors (*n *= 15)	*U* value	*P*-value	Reference range
Platelets (×10³/µL), Median (IQR)	32.0 (20.5-53.5)	38.0 (23.0-53.0]	717	0.834	150-450
Alanine aminotransferase (U/L), Median (IQR)	86.0 (41.7-299)	390.7 (185.5-661.8)	430.5	0.009	05-45
Aspartate aminotransferase (U/L), Median (IQR)	215.4 (77.0-717.5)	1185.0 (552.0-2048.0)	381.5	0.003	0-35
Prothrombin time (seconds), Median (IQR)	16.2 (14.3-18.3)	32.0 (24.7-36.0)	180.5	<0.001	12.3-14.5
Prothrombin time-international normalized ratio, Median (IQR)	1.25 (1.10-1.50)	2.48 (1.94-2.86)	121.5	<0.001	0.8-1.2
Serum lactate (mmol/L), Median (IQR)	3.0 (2.0-4.4)	13.6 (8.8-15.7)	87.5	<0.001	0.5-1.5
Creatinine (mg/dL), Median (IQR)	0.73 (0.60-0.89)	1.05 (0.71-1.30)	489	0.038	0.5-1.0
C-reactive protein (mg/L), Median (IQR)	0.77 (0.26-1.93)	1.29 (0.78-2.48)	569	0.161	0.08-1.12

Performance of individual lab parameters

Correlation Between Key Parameters

Spearman’s correlation analysis revealed significant positive correlations between the key predictor variables. Lactate levels showed moderate-to-strong correlations with both coagulopathy (PT-INR: ρ = 0.585, *P *< 0.001) and hepatic injury (AST: ρ = 0.523, *P *< 0.001). A similarly strong correlation was observed between AST and PT-INR (ρ = 0.522, *P *< 0.001). These findings suggest interconnected pathophysiological pathways involving metabolic acidosis, coagulopathy, and hepatocellular injury in severe dengue (Table 4).

**Table 4:** Spearman correlation matrix of key lab parameters. All correlations are positive and statistically significant at *P *< 0.001. PT-INR, prothrombin time-international normalized ratio; AST, aspartate aminotransferase

Variable pair	Spearman's ρ (rho)	*P*-value
Lactate vs. PT-INR	0.585	<0.001
Lactate vs. AST	0.523	<0.001
AST vs. PT-INR	0.522	<0.001

Predictive Performance

**Figure 1:**
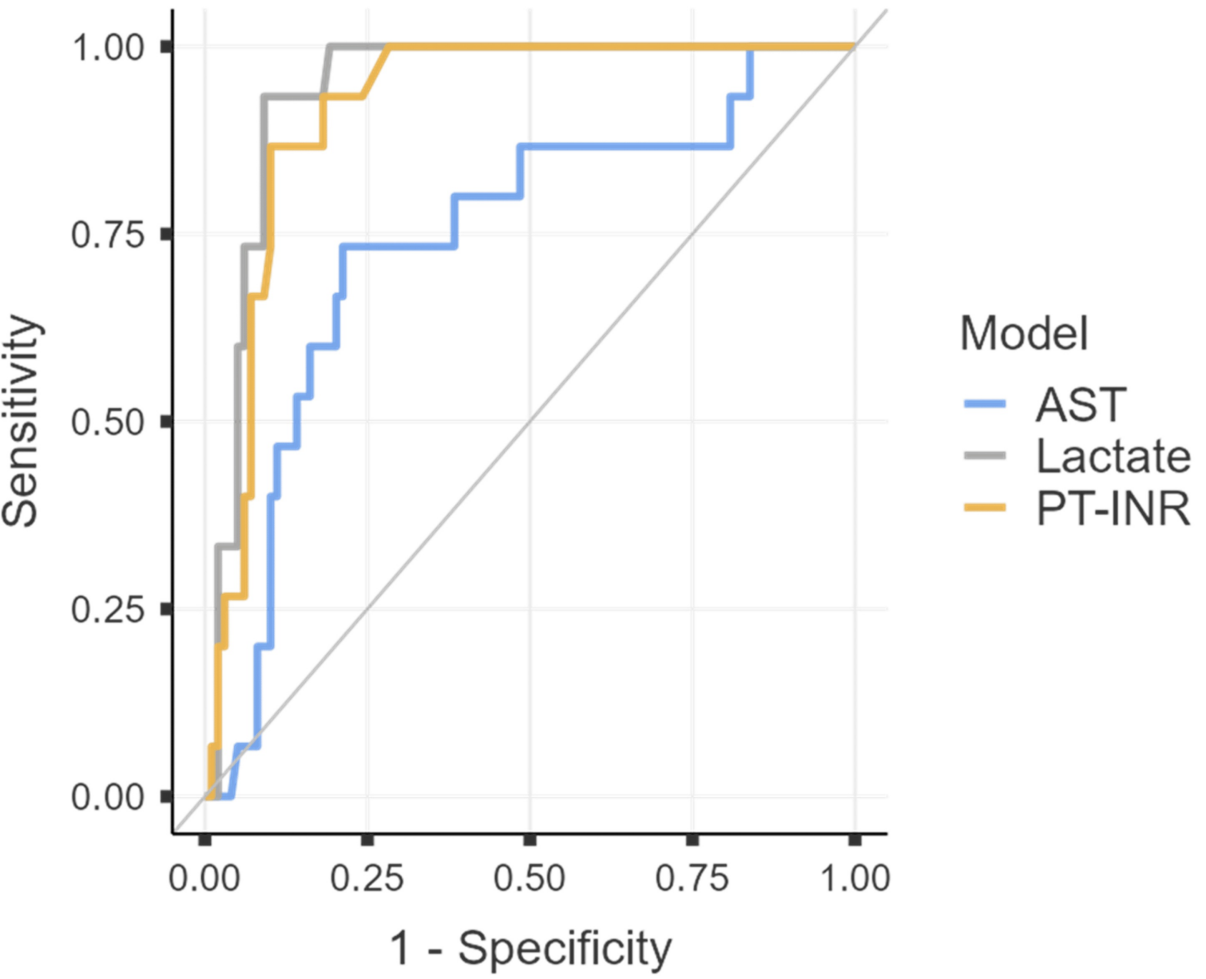
ROC analysis of individual admission laboratory parameters for dengue mortality. Receiver operating characteristic (ROC) curves demonstrating the individual discriminative ability of serum lactate, prothrombin time-international normalized ratio (PT-INR), and aspartate aminotransferase (AST) at admission.

The discriminative ability of AST, PT-INR, and lactate for predicting mortality was assessed using ROC curve analysis. The combined ROC curves are presented in Figure 1. The AUC, optimal cutoff values (determined by Youden’s index), and associated diagnostic accuracy metrics for each predictor are summarized in Table 5.

ROC analysis demonstrated strong individual discriminative ability for lactate (AUC 0.940, 95% CI: 0.897-0.984) and PT-INR (AUC 0.918, 95% CI: 0.865-0.971), with moderate performance for AST (AUC 0.743, 95% CI: 0.602-0.884). Data-driven optimal cutoffs (Youden's index) were: lactate ≥6.6 mmol/L, PT-INR ≥1.855, and AST ≥841.5 U/L.

**Table 5:** Discriminative performance of individual predictors. Cutoff values were determined using Youden’s index. AUC, area under the curve; PPV, positive predictive value; NPV, negative predictive value; CI, confidence interval

Predictor	Optimal cutoff	AUC (95% CI)	Sensitivity (95% CI)	Specificity (95% CI)	PPV (95% CI)	NPV (95% CI)	Accuracy (95% CI)
AST (U/L)	≥841.5	0.743 (0.602-0.884)	73.33% (44.90%-92.21%)	78.79% (69.42%-86.36%)	34.38% (24.35%-46.02%)	95.12% (89.33%-97.85%)	78.07% (69.35%-85.28%)
PT-INR	≥1.855	0.918 (0.865-0.971)	86.67% (59.54%-98.34%)	89.90% (82.21%-95.05%)	56.52% (41.15%-70.74%)	97.80% (92.44%-99.39%)	89.47% (82.33%-94.44%)
Lactate (mmol/L)	≥6.6	0.941 (0.898-0.984)	93.33% (68.05%-99.83%)	90.91% (83.44%-95.76%)	60.87% (45.13%-74.64%)	98.90% (93.12%-99.83%)	91.23% (84.46%-95.71%)

Performance Analysis

All three biomarkers showed significant discriminative ability for mortality (*P* < 0.001). Lactate demonstrated the highest accuracy with an AUC of 0.941 (95% CI: 0.898-0.984), followed by PT-INR (AUC = 0.918, 95% CI: 0.865-0.971). AST showed moderate discrimination (AUC = 0.743, 95% CI: 0.602-0.884). At optimal cutoff points, lactate achieved the highest sensitivity (93.33%) and specificity (90.91%). PT-INR showed strong sensitivity (86.67%) and specificity (89.90%). AST demonstrated lower sensitivity (73.33%) and specificity (78.79%). Pairwise comparisons of ROC curves demonstrated that lactate and PT‑INR both had significantly higher AUCs than AST. The AUC difference between AST and lactate was −0.1980 (95% CI: −0.3424 to −0.0536; *z *= −2.688; *P* = 0.007), and between AST and PT‑INR was −0.1751 (95% CI: −0.3036 to −0.0465; *z* = −2.670; *P* = 0.008). In contrast, the AUCs of lactate and PT‑INR were statistically similar, with an AUC difference of 0.0229 (95% CI: −0.0309 to 0.0767; *z* = 0.834; *P* = 0.404).

## Discussion

This study highlights the utility of basic, universally available lab parameters in identifying pediatric dengue patients at the highest risk of mortality. Our findings demonstrate that admission lactate, PT-INR, and AST possess high discriminative ability for fatal outcomes in dengue. Specifically, the strong intercorrelations observed between these parameters suggest shared pathways involving endothelial dysfunction, microvascular thrombosis, and tissue hypoperfusion. ROC analysis provided the following optimized derivation cutoffs: lactate ≥6.6 mmol/L, PT-INR ≥1.855, and AST ≥841.5 U/L. These thresholds differ from common clinical cutoffs, reflecting severity discrimination in severe dengue rather than general critical care. Lactate emerged as the strongest predictor (AUC 0.940), with a sensitivity (93.33%) and specificity (90.91%) at ≥6.6 mmol/L. This threshold exceeds the 2-4 mmol/L threshold used for benchmarks typically referenced in pediatric sepsis guidelines, suggesting that the mortality risk is associated with significantly higher lactate levels in dengue [12]. PT-INR showed similar discriminative performance (AUC 0.918), highlighting the role of coagulopathy in dengue mortality. An ≥1.855 cutoff indicates moderate coagulation abnormalities with prognostic implications. AST (AUC 0.743) remained relevant, with correlations to lactate (ρ = 0.523) and PT-INR (ρ = 0.522), suggesting the contribution of hepatic injury to metabolic derangement and coagulopathy.

The strong intercorrelations between lactate, PT-INR, and AST support a model in which endothelial dysfunction leads to capillary leakage, tissue hypoperfusion, coagulation activation, and hepatic congestion. This triad may represent the final common pathway in fatal dengue. This study showed that hyperlactatemia, coagulopathy, and hepatocellular injury are strongly associated with mortality in dengue infection. Non-parametric statistical methods address the non-normal distribution of these parameters and provide robust estimates of these associations. Our findings align with severe dengue being a systemic inflammatory condition causing endothelial dysfunction and tissue hypoperfusion [11]. Elevated lactate levels in non-survivors likely reflect a compounding mechanism of anaerobic metabolism (increased production) and impaired hepatic clearance [13]. While non-specific for a single etiology, this dual dependency renders lactate a robust integrated marker of total physiological collapse in severe dengue. The lactate-coagulation correlation suggests shared pathways related to endothelial activation. Hepatocellular injury may result from viral infection, hypoxia, or immune-mediated damage [14]. The multifold elevation in AST levels in non-survivors highlights hepatic involvement in severe dengue. The determinants of pediatric dengue mortality include early warning signs and organ dysfunction, reflecting plasma leakage and multi-organ failure [15]. Laboratory parameters help identify high-risk children, enabling timely interventions. Among PICU patients with severe dengue, cardiorespiratory and hepatic failure were key factors associated with mortality [16]. Early elevated lactate levels indicate poor tissue perfusion despite resuscitation [17].

Clinical implications

For pragmatic bedside application, rounded thresholds of lactate ≥ 6 mmol/L, PT-INR ≥ 1.8, and AST ≥ 800 U/L may be easier to implement, but these values remain exploratory and should not yet be used as definitive triage criteria without external validation. We suggest routine measurement of lactate, PT‑INR, and AST at admission for children with suspected severe dengue, particularly in critical care settings where early escalation decisions are needed. Closer monitoring and proactive management should be considered when values exceed proposed threshold levels or multiple parameters are significantly deranged. Integrating lactate, coagulation, and liver function tests into standard monitoring protocols enables the early identification of high-risk patients, allowing timely care escalation, preemptive management of complications, optimal resource allocation, and earlier intervention considerations. Furthermore, in peripheral healthcare settings, these thresholds could serve as objective criteria for early referral to tertiary care, potentially reducing delays in life-saving interventions.

Limitations

This study had several limitations. First, as a single-center retrospective study, the sample size was limited, which may underpower the detection of modest risk factors (odds ratios < 10). Nevertheless, the effect sizes observed for the primary markers in this cohort were substantial, allowing for robust associative inference and high statistical significance (*P *< 0.001) despite the small sample size. The retrospective design may introduce potential confounding factors. Factors influencing laboratory parameters, such as nutrition, could not be assessed due to limited data capture. Additionally, the relatively small number of mortality events, which is common in dengue studies, constrains the complexity of multivariable models that can be reliably constructed in this study. The statistically optimized cutoffs, while providing maximum discriminative accuracy in our cohort, require prospective validation in diverse settings before definitive clinical implementation. As the study is limited to the single‑center setting in a tertiary pediatric critical care unit, it limits generalizability; the findings may not apply to children managed on general wards, in outpatient settings, or in different epidemiological contexts. While our data reflect a standardized institutional sampling protocol, variations in pre-analytical practices across other centers may influence the generalizability of absolute cutoff values.

Future directions

Despite these limitations, our findings support the inclusion of lactate, coagulation profile, and liver function tests in the initial assessment of dengue patients. However, the proposed risk model is a preliminary derivation, and its promising performance should be considered as hypothesis-generating. Given that this was a single-center derivation study, the immediate priority for future research is the external validation of these cutoff values (lactate >6.6 mmol/L, PT-INR >1.8, AST >800 U/L) in diverse, multicenter cohorts. It will confirm generalizability, calibrate performance, and refine cutoff values before clinical use. Beyond static admission values, further research should explore the dynamic changes (clearance kinetics) of these biomarkers and their response to fluid resuscitation. Further research should explore the dynamic changes in these biomarkers and their responses to interventions. 

## Conclusions

In conclusion, hyperlactatemia (≥6.6 mmol/L), coagulopathy (PT-INR ≥1.8), and hepatocellular injury (AST ≥800 U/L) at admission are strongly associated with mortality in dengue infection, with lactate and PT-INR showing robust discriminative ability. These parameters, given their intercorrelations, may help identify high-risk patients early. While integrating these biomarkers into dengue protocols could enhance risk stratification, these findings require prospective external validation across diverse populations to confirm generalizability and assess real-world performance before definitive clinical implementation.
